# The Developing Brain in the Digital Era: A Scoping Review of Structural and Functional Correlates of Screen Time in Adolescence

**DOI:** 10.3389/fpsyg.2021.671817

**Published:** 2021-08-27

**Authors:** Laura Marciano, Anne-Linda Camerini, Rosalba Morese

**Affiliations:** ^1^Institute of Public Health, Università della Svizzera italiana, Lugano, Switzerland; ^2^Faculty of Biomedical Sciences, Università della Svizzera italiana, Lugano, Switzerland; ^3^Faculty of Communication, Culture and Society, Università della Svizzera italiana, Lugano, Switzerland; ^4^Department of Business Economics, Health and Social Care, University of Applied Sciences and Arts of Southern Switzerland, Manno, Switzerland

**Keywords:** adolescence, brain, fMRI, cognitive control, reward, media effects

## Abstract

The widespread diffusion of screen-based devices in adolescence has fueled a debate about the beneficial and detrimental effects on adolescents’ well-being and development. With the aim of summarizing the existing literature on the associations between screen time (including Internet-related addictions) and adolescent brain development, the present scoping review summarized evidence from 16 task-unrelated and task-related neuroimaging studies, published between 2010 and 2020. Results highlight three important key messages: (i) a frequent and longer duration of screen-based media consumption (including Internet-related addictive behaviors) is related to a less efficient cognitive control system in adolescence, including areas of the Default Mode Network and the Central Executive Network; (ii) online activities act as strong rewards to the brain and repeated screen time augments the tendency to seek short-term gratifications; and (iii) neuroscientific research on the correlates between screen time and adolescent brain development is still at the beginning and in urgent need for further evidence, especially on the underlying causality mechanisms. Methodological, theoretical, and conceptual implications are discussed.

## Introduction

Today’s adolescents have grown up in the digital era. More than any generation before, their life has been shaped by the constant availability of online contents and services, the 24/7 possibility to reach and be reached by others, and the easy access to gratifying and personalized contents and functionalities on screen-based devices. The widespread diffusion of screen-based devices in the adolescent population, including laptops, tablets, and, particularly, smartphones, has raised concerns about detrimental clinical and psychological effects of excessive screen time (Domingues-Montanari, 2017), defined as the amount of time spent interacting with screens in a specific period. However, according to a recent review of eighty reviews (Orben, 2020), the existing literature on screen time and well-being is characterized by considerable heterogeneity, with most of the studies relying on cross-sectional, self-report, and low-quality data. In general, the relationship between any form of screen time, including social media use, and well-being proved to be negative but small, and more rigorous and better-designed research is now urgently needed to provide solid evidence. Considering the limitations highlighted in previous reviews, it is now pivotal to look directly at the core structure involved in the use of screen media and their effects: the brain. During the last two decades, reviews of neuroimaging studies were conducted to investigate the relationship between (excessive) screen time and brain functioning (Kuss and Griffiths, 2012; Brand et al., 2014, 2016, 2019; Meng et al., 2015; Cerniglia et al., 2017; Yao et al., 2017; Crone and Konijn, 2018). However, only two of them included the adolescent population, but they were based on a narrative – and not systematic – approach. While Cerniglia et al. (2017) focused on the description of adolescents with Internet addiction, including prevalence rates, clinical assessment, and types of intervention, Crone and Konijn (2018) described different preliminary perspectives through which social media may impact adolescents’ development, including reactions to online peer exclusion and acceptance, the online influence of peers, and emotion regulation. Another three reviews made use of a systematic approach (Kuss and Griffiths, 2012; Meng et al., 2015; Yao et al., 2017) to summarize studies on Internet addiction and Internet Gaming Disorder adult samples. Furthermore, Brand et al. (2014, 2016, 2019) proposed the Person-Affect-Cognition-Execution (I-PACE) model to outline the psychological and neurobiological mechanisms behind the development and maintenance of addictive online behaviors, but without conducting a systematic literature review of the existing studies and with no specific focus on the adolescent brain. Yet none of them considered newer forms of screen media (e.g., smartphone use). At the same time, the majority of previous reviews summarized results on different neuroimaging techniques (i.e., EEG, PET, SPECT, MRI, and fMRI), and tasks transposed to the online world (e.g., online peer rejection).

Although the literature on the associations between screen media use and adolescent brain development is still in its infancy, a more holistic and systematic summary on available research findings to date is now crucial to draw a comprehensive picture of what has been already investigated and where are the gaps to be filled with future research. Hence, the present scoping review is now pivotal (1) to systematically examine the emerging evidence of the relationship between screen time and adolescent brain development and to report on how research is conducted on this topic, and (2) to identify research gaps and thus highlight new and vital routes of research, giving specific guidance for future works (Meshi et al., 2015; Munn et al., 2018). To facilitate a critical appraisal of the neuroimaging studies conducted on the topic, the present scoping review focuses on studies using the functional magnetic resonance imaging (fMRI) approach. This neurophysiological method can capture the complex neuronal changes, which may occur in adolescents who spend a lot of time with screen devices and show problematic usage behaviors (Meng et al., 2015; Yao et al., 2017; Sharifat et al., 2018).

### The Adolescent Brain

Adolescence, defined as the transition from childhood to adulthood (Sawyer et al., 2018), is a developmental period in which brain regions undergo significant changes influenced by biological and environmental factors (Burnett et al., 2011; Larsen and Luna, 2018). In general, cognitive abilities promoting effective self-regulation have been reported to grow gradually during adolescence, together with neural correlates related to efficiency in information-processing, e.g., axonal myelination, and higher-order cognitive functions, including, among others, the prefrontal cortex (PFC), anterior cingulate cortex (ACC), and parietal regions (Steinberg, 2008; Albert et al., 2013; Caballero et al., 2016). Initially under-developed, the cognitive-control system matures progressively, and it augments adolescents’ ability to self-regulate their behaviors, especially their emotions (Albert et al., 2013; Casey and Caudle, 2013). In particular, the cognitive/cold and the affective/hot control systems are associated with different but interrelated sub-regions of the PFC, i.e., the dorsolateral-prefrontal-cortex (DLPFC) and the orbitofrontal/ventromedial prefrontal cortex (OFC/VMPFC), respectively. The early maturation of the cognitive control system drives the maturational process of emotion regulation during adolescence (Schweizer et al., 2020). In particular, increased emotion regulation depend on the augmented connectivity of prefrontal brain regions to the amygdala and the striatum, regions implicated in emotion and reward processing (Aldao et al., 2016). The affective-motivational system also changes in relation to pubertal hormones, with diverse effects of androgens and estrogens on brain structures, including subcortical brain regions related to emotion-processing, sensitivity to social and emotional stimuli, motivation, and gratification (i.e., the amygdala, hippocampus, striatum including the nucleus accumbens-NAcc, caudate, putamen, and globus pallidus) (Goddings et al., 2014). The earlier maturing affective-motivational system is also related to increased dopaminergic activity, with new projections from mesolimbic to prefrontal areas. The parallel development of different brain areas, i.e., the frontoparietal and subcortical structures, posits adolescence as a particular period in which there is an imbalance in brain development, better described by the “dual-systems model” (Steinberg, 2010). According to this developmental model, the affective-motivational system matures in early adolescent years with respect to the control system, which reaches maturity in young adulthood. The temporal gap between the maturation of the two systems creates a period of greater vulnerability and propensity to risk-taking as well as reward- and novelty- seeking behaviors during middle adolescence (Willoughby et al., 2014), especially when a social component is involved (Galván, 2013). At the same time, adolescents are still not fully able to respond adequately with their behavior to positive and negative situations, which is a capacity that develops in later years alongside an increasing strength in cortical-subcortical connectivity linked to better cognitive performance (van Duijvenvoorde et al., 2016) and to an increased ability to evaluate positive and negative emotional consequences of one’s behaviors (Johnson et al., 2008).

### Screen Time in Adolescence

During adolescence, the time spent with parents, and parental influence in general, declines, while peers become more relevant (Steinberg, 2002). In search for more independence, adolescents prefer to stay with their friends or alone (Dijkstra and Veenstra, 2011). The Internet, accessible through different screen-based devices, provides adolescents with many opportunities to “escape” from parents, and everyday problems in general, to connect with peers (e.g., through instant messaging and social media applications such as WhatsApp, Messenger, Instagram, and Snapchat), or to simply engage in highly gratifying activities (e.g., listening to music, watching videos, online gaming). As such, the Internet plays a crucial role during this developmental period (Crone and Konijn, 2018).

Online communication and entertainment activities are particularly relevant for adolescents’ psychosocial autonomy, which is promoted by the development of self-identity and by the capacity to initiate and maintain meaningful relationships with others (Steinberg, 2002). Compared to childhood, adolescence is characterized by more intricate and hierarchical peer relationships with larger social networks, also called crowds (Garner et al., 2006). Crowds typically promote their own values, including original dressing, talking, and behavioral styles. In order to be part of these crowds, adolescents feel pressured to act accordingly (Dijkstra and Veenstra, 2011). Not surprisingly, they also experience hypersensitivity to peer acceptance and rejection (Somerville, 2013). Indeed, crowds may affect adolescents’ self-esteem, induced by social comparison and social norms (Steinberg, 2002). In this regard, interactions via social media or instant messaging applications were found to supplement the face-to-face flow of information in a significant way (Van Cleemput, 2010).

Peer influence is not only relevant for the sense of the self, but also for the engagement in risky behaviors. During adolescence, most individuals start to consume alcohol, tobacco, or illicit drugs, have their first sexual experiences, engage in violent (online) activities, and tend to break the rules more often (Steinberg, 2002). The likelihood of being involved in such behaviors is higher under the influence of peers, since their presence triggers a motivational state in which immediately available rewards are more valued than long-term rewards, thus augmenting the tendency to seek short-term gratifications deriving from risky choices (Albert et al., 2013). According to a meta-analysis by Vannucci et al. (2020), the consumption of social media in adolescence is positively related to more risky behaviors, such as substance use and unprotected sex, since they all share the same nature of rewards. Furthermore, the authors conclude that the association among these behaviors is stronger in younger adolescents.

In line with the dual-system model, it is not surprising that adolescents spend a lot of time with screen devices to consume entertainment contents or communicate with others (Camerini et al., 2020a). Oftentimes, they use digital devices without considering the potential risks of engaging in specific online activities or spending excessive time with these devices, simply because their brains are tuned to do so. Through screen-based activities, adolescents engage in particularly rewarding behaviors (Olsen, 2011). Reactions to rewards are a key aspect of the adolescent brain, since, compared to children and adults, their neural responses to environmental stimuli are more pronounced and sustained (i.e., more dopamine release), especially when stimuli involve social interaction (Galván, 2013). This is in line with the monotonic decrease in connectivity between the NAcc and VMPFC, which happens during adolescence and reflects an increased pleasure for gratifying contents (van Duijvenvoorde et al., 2016).

Furthermore, adolescents use the smartphone and social media to “kill” time, keep in touch, and stay up to date (Throuvala et al., 2019; Allaby and Shannon, 2020). A repeatedly identified driver of smartphone and social media use is the fear of missing out (FoMO) (Elhai et al., 2018). FoMO is defined as “a pervasive apprehension that others might be having rewarding experiences from which one is absent” (Przybylski et al., 2013, p. 1841). The resulting urge to constantly check online contents and the incoming notifications have negative consequences on adolescents’ cognitive development and academic achievement (Dontre, 2020), also because online checking behaviors distract during study time, interfere with ongoing activities, and limit cognitive processing capacities (van der Schuur et al., 2015; Camerini and Marciano, 2020). Furthermore, the prolonged use of screens, especially during night hours, was found to be associated with later onset of sleep and reduced sleep quality (Hale and Guan, 2015; Carter et al., 2016), which, in turn, have negative consequences on the brain functioning (Telzer et al., 2013).

### Problematic Screen Time in Adolescence

With “problematic” screen time, the present review summarizes different online behavioral addictions, including Internet, social media, and smartphone addiction (Block, 2008; Shaw and Black, 2008; Kwon et al., 2013; Zhitomirsky-Geffet and Blau, 2016; Lin et al., 2017; Chin and Leung, 2018; Jo et al., 2019). Adolescents with online behavioral addictions generally present one or more of the following characteristics: cognitive salience, mood modification, tolerance, withdrawal symptoms, conflict, and relapse (Jo et al., 2019). Cognitive salience refers to the propensity to think of the Internet (and more specific online activities) event when not spending time on it. Mood modification comprises being online as a coping strategy in stress-related situations. Tolerance involves the need to spend increased time online to experience the same amount of gratification as before. Withdrawal embraces anxiety, distress, and irritability symptomatology, including the urge to check online contents when one is not able to use the Internet. Conflict comprises problems with other people (e.g., family and friends) as well as at work/school, sports, and other hobbies due to excessive online activities. Relapse denotes the propensity to fall back in problematic usage behaviors after periods of non-use.

According to the Interaction of Person-Affect-Cognition-Execution (I-PACE) model (Brand et al., 2016, 2019), cognition, personality, existing psychopathology, and motivations determine how people use the Internet in a specific way. The behavior is the crucial component to consider, whereas the type of environment (online versus offline) may be of secondary importance, although the environment contributes significantly to the manifestation of addictive behaviors. The cognitive and affective response to Internet use, together with the gratifications obtained, foster its subsequent use. At the same time, lower efficiency of neural systems related to impulsivity, like the PFC, fails to inhibit the control over personal urges. In their early stages, specific online behaviors may provide gratifications and relief from negative moods (Laier and Brand, 2017), thus creating reward expectancies and modifying individual coping styles. Repetitive checking of online contents and the gratifications obtained, act as a reinforcement for the user (Oulasvirta et al., 2012). In a subsequent stage, conditioning processes lead to compulsive use, thus incrementing problematic online behaviors. The imbalance between growing gratifying-oriented urges and diminishing inhibitory control over these urges are pivotal for the development and maintenance of online behavioral addictions.

Connectivity alterations in structures included in frontostriatal circuits were related to emotion dysregulation in other addictive disorders (Wilcox et al., 2016), and the frontostriatal dysfunction is thought to promote a compulsive use of the Internet and screen devices in general (Feil et al., 2010; Lüscher et al., 2020). In line with assumptions of the I-PACE model, two meta-analyses focused on the neural changes in individuals with Internet Gaming Disorder, also called Online Gaming Disorder (Meng et al., 2015; Yao et al., 2017). They reported neural abnormalities in frontostriatal and fronto-cingulate circuits as well as dysfunctions in the prefrontal lobe in individuals with this disorder, now recognized by the Diagnostic and Statistical Manual of Mental Disorders (American Psychiatric Association, 2013) (DSM-5). Notably, in light of the dual-system model of the adolescent brain, a prominent sensitivity to gratifying (social) contents and a greater affective response, which is not completely modulated by the cognitive control system of OFC/VMPFC, likely lead to higher levels of problematic Internet use.

## Research Objectives

The present paper aims to systematically summarize the existing literature on neural correlates of screen time (including problematic screen time) in adolescence, via a scoping literature review. It considers the full range of the developmental period between pre-adolescence and late adolescence. To the best of our knowledge, this is the first review, carried out with a systematic approach, focusing on the role of screen devices in the development of the adolescent brain. This scoping review concentrates on the two main neuroimaging approaches: the task-unrelated and the task-related paradigm. Functional connectivity allows examining functional interactions between brain regions by studying the temporal dependency between anatomically separated brain areas (van den Heuvel and Hulshoff Pol, 2010). In particular, the brain regions that, in the resting state, show a spontaneous interaction are termed as resting-state networks (Heine et al., 2012; Lee et al., 2013) (RSNs). RSNs have been defined by their configurations and functions, i.e., driven by internal processes and external processes. The first category includes the default mode network (DMN), the central executive network (CEN), and the salience network (SAL). On the other hand, external processes are related to networks, including specialized functions, such as the auditory (AN), visual (VN), and sensorimotor (SMN) networks (Lee and Frangou, 2017). In addition, the present scoping review also includes two specific MRI methods that are applied to study gray and white matter microstructure in relation to screen time in adolescence: the voxel-based morphometry (VBM) and the diffusion tensor imaging (DTI). VBM is a neuroscientific approach that allows highlighting brain differences using structural MRI. It is sensitive to both global and local differences in gray and white matter, and it was used to study brain differences both in clinical and healthy subjects (Mechelli et al., 2005). In addition, DTI was used to investigate microstructural changes in the diffusion of water molecules of white matter, before they can turn into a macro-structural loss of white matter (detected by the VBM). Using DTI, different characteristics of water molecules diffusion can be extracted, providing more details on tissue microstructure. In particular, the Fractional Anisotropy (FA) and Mean Diffusivity (MD) are two widely used indices to measure white matter integrity, and they describe, respectively, the coherence and the mean diffusion of molecules diffusivity in white matter. Hence, a decrease in FA (a loss of anisotropy), and an increase in MD (an augmented diffusion due to WM degeneration) are indicative of a deficit in WM microstructure. Also, DTI imaging was used to determine subtle abnormalities in diverse diseases, and it is currently part of clinical assessments (Bihan et al., 2001). Both the VBM and the DTI imaging approach are also considered in the present review of resting-state fMRI studies.

Task-based fMRI allows understanding the brain areas activated during the performance of a specific task or in response to a stimulus compared to a baseline condition (Lee et al., 2013). Using the blood-oxygen-level-dependent (BOLD) signal, which reflects the synaptic activity of local field potentials underlying cognitive processes, fMRI allows examining the neural correlates of specific (media-related) tasks in adolescents (Ekstrom, 2010).

## Methods

### Literature Search

The scoping review was conducted according to the corresponding guidelines (Peters et al., 2015). The literature search was carried out on March 31, 2020, in the following eight academic databases: Communication & Mass Media Complete, Psychology and Behavioral Sciences Collection, PsychInfo, and CINAHL (all via Ebscohost), ERIC and Proquest Sociology (via Proquest), Medline (via Pubmed and Proquest), and Web of science. Different keywords in titles and abstracts related to the intervention (e.g., screen time, smartphone use), outcome (e.g., brain, fMRI), and population (e.g., adolescent) were used in the research string (see Appendix for the complete list of keywords). An additional hand search was carried out in the first 100 entries of Google Scholar, using the same keywords. Only studies published in peer review journals between 2010 and 2020 were considered in this review. All entries were imported in Zotero to aid the removal of duplicates.

### Study Selection

After duplicates, book chapters, theses, and conference papers were removed, the first two authors independently carried out the title and abstract screening. To be included in the present review, a study had to: (1) be written in English, (2) published in a peer-review journal, (3) include an underage population (mean age ≤ 18 years), with (4) no comorbid psychiatric diagnosis (e.g., suicidal ideation, ADHD, depression, gambling) with the exception of Internet addiction or problematic Internet, smartphone and social media use, (5) include original research results of MRI, fMRI, or VBM analyses. In addition, studies were excluded if they focused on Internet gaming disorder (for an overview of neuroscientific correlates of the latter see 7 and 8) or if they focused on clinical interventions such as cognitive-behavioral therapy, cognitive training, or brain-computer interface studies. Cohen’s kappa statistic for the title and abstract screening was obtained as a measure of inter-coder reliability. Discrepancies at each screening stage were solved through a consensus meeting with the third author.

### Data Extraction

For each included study, the following information was extracted: first author, year of publication, journal title, the country where the study was conducted, study design (cross-sectional or longitudinal), type of sampling, characteristics of the sample (including sample size, % of male, age, screening for psychiatric diagnosis), the type of screen-based activity investigated in the study, the measure used to assess the construct(s) under investigation (including if it was done by self-report or trace data), the specific task(s) (applied in fMRI studies), studied outcomes of brain activity, and a brief description of the results. Summary tables (Appendix Tables 2, 3) of all the studies are reported in the Appendix.

## Results

### General Overview

The initial search returned a total of 2785 entries. After removal of 1191 duplicates, 26 book chapters, and 19 theses, 1549 publications were retained for the title and abstract screening. Of these, 1533 were excluded. Cohen’s kappa for the title and abstract screening was 0.762, indicating a substantial level of agreement (McHugh, 2012). During the full-text screening of the remaining 16 publications, two were excluded because they included participants with more than 18 years of age. On the contrary, two additional studies were added after the hand search, resulting in 16 publications considered in this scoping review.

All studies were published between 2011 and 2020, reflecting the recency of this field of research. North America and Asia were the most represented continents with five studies conducted with adolescent populations from the U.S., five from China, four from Korea, one from Japan, and one from Europe (Netherlands). Nine studies were controlled trials, five used a cross-sectional design, one used a randomized experimental design, and another one included both a cross-sectional and a longitudinal design collecting data over 36 months. Sample sizes ranged from 12 participants (Hong et al., 2013b) to 2532 (Rodriguez-Ayllon et al., 2020), with a minimum age of 10 and a maximum age of 18 years. Four studies investigated neural correlates of screen-based activities, and three focused on social media activities (i.e., receiving Likes). Concerning problematic screen time in adolescence, eight studies focused on IA and one on excessive smartphone use. In the next sections, the studies are briefly summarized, in groups of similar concepts, by differentiating studies focusing on non-clinical versus clinical samples, and by distinguishing different applied methodologies.

### Screen Time in Adolescence

#### Task-Unrelated Paradigm

Three studies investigated the effect of screen time on the developing brain of adolescents in the general population. Two of them (Takeuchi et al., 2018; Rodriguez-Ayllon et al., 2020) focused on gray and white matter changes associated with the frequency and duration of screen time. In particular, Rodriguez-Ayllon et al. (2020) collected data through parent-report on the amount of television, computer, and video games use in a large sample (*n* = 2532) of 10-year-old participants, together with DTI data. Controlling for sex, age, ancestral background, Body Mass Index, maternal education, emotional and behavioral problems, and non-verbal Intelligence Quotient (IQ), screen time was not significantly associated with any global DTI metrics (including FA and MD). In other words, screen time was not related to white matter microstructure abnormalities, thus supporting previous research indicating that the relationship between screen time and mental health outcomes are null or very small (Orben and Przybylski, 2019). Contrary, physical activity, particularly sports participation, was positively associated with FA. Sports participation, together with outdoor playing, was also negatively associated with MD. Interestingly, associations between physical activity and white matter were still present after adjusting for levels of screen time. This indicates that the benefits of sports participation and outdoor play are not neutralized by spending time in front of screens. This result supports previous findings on the positive role of physical activity on neuropsychological performance and cognition, since it may improve cerebral blood flow and decrease cardio-metabolic risk factors (Prakash et al., 2015).

The study by Takeuchi et al. (2018) included cross-sectional as well as longitudinal data on the effect of the frequency of Internet use on gray and white matter integrity in a sample of 284 early adolescents aged 11 years. Using VBM, the authors did not find any correlations between regional gray and white matter volumes and the frequency of Internet use at cross-sectional levels. However, longitudinal analyses with data collected from study participants aged around 14 years, and corrected for confounding variables (including age), revealed that the frequency of Internet use negatively predicted change (i.e., smaller volume increase) in regional gray and white matter volumes of a widespread anatomical cluster (including temporal – perysilvian regions, medial temporal – hippocampus and amygdala, prefrontal – including the OFC, insula, and cerebellar structures), and that change spread to the adjacent white matter areas, including regions in the cingulate cortex. The mentioned brain areas are related to language processing, attention and executive functions, emotions, and reward. In addition, the frequency of Internet use significantly and negatively correlated with the change in verbal IQ. A decrease in verbal intelligence measured by the Wechsler IQ test (Wechsler, 1949), which involves verbal skills, knowledge, attention, and working memory, can be associated to a smaller development in the reported regions caused by frequent Internet use (e.g., lower hippocampal activity and memory performance). Furthermore, it is possible that participation in online activities, including highly rewarding stimuli, may be related to desensitization to general reward and difficulty in enjoying pleasure, thus deflecting mood and augmented impulsivity, as highlighted by the involvement of the OFC.

In the study by Horowitz-Kraus and Hutton (2018), parents of nineteen 10-year-old participants reported on their children’s time spent reading for fun and general information about screen time, measured as the time passed on the smartphone, tablet, laptop, and television. The time that pre-adolescents spent with screen devices negatively correlated with functional connectivity between the visual word form area and the regions related to language, visual processing, and cognitive control, whereas time spent reading for fun positively correlated with these regions. The results highlight the neurobiological benefits of reading, which involves different cognitive tasks like sustaining attention, visualizing, and imagining what was described in a story. At the same time, the results suggest a possible negative effect of screen time on the pre-adolescent brain, which can be associated to a delay in language acquisition and academic problems, possibly related to a diminished verbal interaction with others and lower capabilities of sustaining the cognitive load of more demanding tasks. More precisely, screen time was associated with reduced connectivity in areas involved in cognitively demanding tasks (such as the ventral ACC, the insula, the VMPFC, and inferior frontal gyrus – IFG) with the visual word form area.

To summarize, the frequency of screen time is only weakly or not related to adolescent brain development, at least cross-sectionally. Given the limited number of studies in this area, the results should be replicated not only in early-to-mid but also late adolescent populations. Such studies should use more detailed measures of screen time (e.g., including smartphone and social media use). Furthermore, more longitudinal research is needed to identify structural changes related to Internet-related habits and use over time (Rodriguez-Ayllon et al., 2020).

#### Task-Related Paradigm

Four studies used a task-based fMRI approach to investigate the effects of different media-related tasks on the adolescent brain.

The study by Sherman et al. (2016) was the first to replicate social media interactions in the MRI setting. The authors developed a novel functional fMRI paradigm to simulate a popular social network, i.e., Instagram. A total of thirty-four adolescents between the ages of 13 and 18 years underwent an fMRI examination while viewing pictures posted on Instagram. The authors investigated behavioral and neural responses during the observation of pictures with different numbers of Likes as a popular sign of approval and appreciation in social media settings. The fMRI task was composed of 148 unique photos, each with their number of Likes, divided into 42 images demonstrating risky and deviant behaviors (e.g., cigarettes, smoking, alcohol, marijuana, paraphernalia, rude gestures), 66 neutral images (e.g., friends, food) taken from other adolescents’ profiles, and 40 pictures of their own Instagram profile. Both kind of pictures, neutral images and images presenting risky or deviant behaviors, were assigned with a popular value of 23 to 45 Likes and an unpopular value of 0 to 22 Likes. The popular values were assigned by the research team during the creation of the fMRI task, though each participant was told that they were the Likes collected from previous participants. During the scan, participants were asked to look at the images and decide whether to select Like using the same criteria as in real life. Results of the fMRI indicated that neural responses are determined by the number of Likes. In particular, all participants showed increased brain activity for pictures with more Likes in all experimental conditions. Significantly greater neural recruitment of the left frontal cortex until the precentral gyrus was found when they viewed pictures with risky and deviant contents that received many Likes compared to few Likes. Furthermore, fMRI results showed that viewing pictures with many Likes was mainly associated with the recruitment of the reward network (e.g., NAcc, caudate, ventral tegmental area). The authors suggested that, when adolescents viewed pictures of risky and deviant behaviors, activation in the cognitive-control network decreased, and this activation was also based on the popularity of the picture in terms of the number of Likes. Therefore, the decrease in cognitive control may reflect the mechanism implicated in getting involved in at-risk situations.

Subsequently, Sherman et al. (2018a) applied the same fMRI Instagram task that used in the previous study (Sherman et al., 2016) to a sample of 34 17-year-old high school students and 27 19-year-old university students. They administered to each participant three experimental conditions based on the picture categories: neutral (e.g., pictures of people, food), risky (e.g., alcohol and partying behaviors, smoking), participants’ own pictures (selected from their Instagram profile but without risky contents). All pictures were defined in a popular version with many Likes (23–45) and an unpopular version with few (0–22) likes. After the magnetic resonance examination, the participants completed a questionnaire of risky events (Katz et al., 2000) (CARE-R). fMRI results showed that, when they received many Likes compared to few, high school and college students did not significantly differ in activation in the left or right NAcc. With this trend, they did not differ in NAcc activation when participants looked at popular (compared to unpopular) risky or neutral images. Instead, during the view of popular than unpopular risky images, college students activated significantly the left NAcc.

The authors investigated the neural correlates of Likes on the reward network in late adolescents’ brain, and found that reward brain system was recruited in response to the popular pictures (i.e., with many Likes). They also tested whether the NAcc response to receiving social approval (in terms of more Likes) for participants’ own pictures was correlated with age. The results of the correlation analysis showed that the response in the left NAcc was significantly higher for university students compared to high school students.

Additionally, Sherman et al. (2018b) investigated the brain areas involved in Liking other people’s picture during a simulation of Instagram activities. Fifty-eight participants, aged 18 years, were scanned in the fMRI setting. The authors used the fMRI task applied in their previous study. For each of the 148 pictures, participants were asked to “like” it or not. After the fMRI examination, they completed a short survey on their opinions about other people’s pictures and whether their decisions were based on their instincts or thoughtful thinking. fMRI results showed the recruitment of neural correlates involved in reward processes (e.g., striatum and ventral tegmental area -VTA) when participants provided Likes to others; additionally, the same brain areas were involved during the experience of receiving Likes from others. Furthermore, authors suggested that providing Likes on Instagram correlates with the ventral striatum and VMPFC, generally involved during reward processing and prosocial behavior.

Very recently, Efraim et al. (2020) investigated in thirty-two children, aged 9 years, if after-school sedentary screen time can model the neural correlates of reward and cognitive control, using a task with pictures of high- and low-calorie food. The fMRI acquisition followed two counterbalanced treatment conditions. The conditions were defined as active and sedentary, and involved activities that took place after school. Each condition, i.e., sedentary (e.g., video games, film) or active play (e.g., beanbag toss, basketball, walking), lasted for 3 h. After each condition, participants underwent MRI examination while performing the Go/No-Go response inhibition task, which included images of high (e.g., candy, ice cream) and low-calorie food (e.g., vegetables, fish). Participants were asked to press a button when they saw an image of low-calorie food, and not to press a button for images of high-calorie food. fMRI results showed no main effect for the “active” condition compared to the “sedentary” (screen time) condition. Based on an *a priori* Region-of-Interest (ROI) analysis, the results showed significant activity of the right superior parietal cortex and the left ACC when participants saw images of high-calorie food, and this activation was higher in the condition of after-school sedentary screen time. The authors concluded that sedentary screen time is associated with a decreased impulse control.

### Problematic Media Use in Adolescence

#### Task-Unrelated Paradigm

Neuroscientific research also focused on structural changes related to problematic Internet use. In a study with 33 17-year-old participants, Zhou et al. (2011) compared gray matter density of Internet addicts (*n* = 18) and controls (*n* = 15). The authors found that the Internet-addicted group had lower gray matter density in the left ACC and PCC, left insula, and left CG. However, no differences were found with regards to white matter integrity. The regions with reduced gray matter are linked to emotional and behavioral problems, often reported by adolescents with high levels of IA. In particular, the anterior cingulate region is involved in motor control, cognition, and arousal, whereas the PCC is implied in visual-spatial and sensorimotor processes, and in self-referential functions related to the DMN. Overall, problems of these functions can be related to a diminished individuals’ ability in monitoring and inhibiting inappropriate behaviors. Since the insula integrates an interoceptive state, conscious feelings, and risk decision-making, it has also been associated with the sensation of urge and the motivational drive to engage in other substance-related addictions (Naqvi and Bechara, 2010).

In addition, Hong et al. (2013a), collected data on the cortical thickness of 15 male adolescents identified as Internet addicts and fifteen controls, ranging from 13 to 15 years old. The authors found a reduction in OFC thickness in the former group. Their result supports other studies on addictions investigating the role of the right OFC in such disorders. This region has been related to decision-making, especially in rewarding situations. In particular, the medial part of the OFC has been investigated in relation to immediate rewards, and the lateral OFC to rewards delay and response suppression as well as to cognitive flexibility and compulsive behaviors.

Lin et al. (2012) collected DTI data on 17 17-year-old adolescents with Internet addiction and 16 controls. Compared to the latter group, Internet addicts had significantly reduced FA in many white matter tracts, such as orbitofrontal tracts, corpus callosum, front-occipital fasciculus, anterior cingulum, corona radiata, internal and external capsule, and tracts of the precentral gyrus. The widespread deficit in white matter integrity reflected a disruption in the organization of white matter tracts in these adolescents. Volume-of-Interest (VOI) analyses showed that decreased FA was mainly due to an increase in radial diffusivity, which reflects destructed axons myelination. It is important to remember that the OFC has wide links to diverse areas, including the prefrontal, visceromotor, and limbic regions, as well as to associative cortices, and it is crucial for emotional- and addictive-related phenomena, such as craving, compulsive-like behaviors, and deficits in decision-making. Decreased FA in the ACC may mirror impaired cognitive control. Altered connectivity in the corpus callosum, corona radiata, and external and internal capsula is typical of other addictions, too, and the latter may echo alterations in fronto-subcortical circuits since it includes connections between the thalamus/striatum and frontal regions, engaged in rewarding and emotional stimuli.

Four studies (Hong et al., 2013b; Wee et al., 2014; Wang et al., 2017; Chun et al., 2018) focused on the functional connectivity of different RSNs and compared adolescents with symptoms of Internet addiction to control subjects. In general, findings point toward decreased functional connectivity in many brain networks, including DMN and CEN. For example, Hong et al. (2013b) reported a general decrease in connectivity in the Internet-addicted group (*n* = 12) of both short- and long-range connections in a network comprising 59 links in 38 different brain regions, compared to the control group (*n* = 11). The larger part of reduced connectivity was found between the subcortical (i.e., hippocampus, globus pallidus, and putamen), frontal, and parietal areas. However, there was no correlation between functional connectivity in the identified networks and the participants’ score on a validated, self-report Internet addiction scale. These results reflect the presence of a cortico-subcortical pathology in Internet addiction, which is in line with other results on addictive behaviors (Lüscher et al., 2020). In particular, once an addiction has been established, compulsive behaviors aimed to seek gratifying stimuli are associated with a loss of prefrontal cortical top–down control over the striatal mechanisms, especially in the putamen, which eventually causes higher engagement in addictive and compulsive habits. Interhemispheric differences were interpreted by the authors either as a vulnerability factor for or as a neural correlate of Internet addiction. Since diminished functional connectivity between frontal and parietal regions, crucial for cognitive control, is common across different types of addictions, it may be a characteristic phenotype which is not merely associated to substance use but also relevant in online behavioral addictions. Interestingly, the widespread decrease of functional connectivity was not related to damages in brain functional network topology. This result demonstrates that the topology and strength of connectivity are two distinct characteristics, and deficits in one do not inherently include deficits in the other. The authors suggested that altered striatal dopamine transporter and D2 receptor availability in the group of Internet-addicted pre-adolescents may be a crucial element in damaging functional connectivity. These alterations have also been found in Internet-addicted adults (Kim et al., 2011; Hou et al., 2012).

In a study involving 17 17-year-old participants with symptoms of Internet addiction and 16 control subjects, Wee et al. (2014) reported network alterations in different brain areas, using a graph theoretical analysis. Through this technique, the authors investigated the functional organization of the brain, which, during the adolescent years, should evolve toward greater functional integration. In particular, functional brain networks are re-organized from local to more distributed networks during brain development, leading to weaker short-range functional connectivity and stronger long-range functional connectivity. The authors found that participants in the Internet-addicted group showed altered networks (i.e., nodal centrality alterations) in the left inferior parietal lobule (IPL) and the right anterior cingulate gyrus (ACG), which are part of the DMN. The DMN generally includes functional links among the posterior cingulate cortex (PCC)/precuneus as well as the medial frontal and inferior parietal regions. Altered networks and dysfunctions in the DMN were also found in studies on other addictive behaviors (Zhang and Volkow, 2019). In addition, in the study by Wee et al. (2014), network alterations included the left thalamus and right middle cingulate gyrus (MCG). Notably, the right ACG and MCG and the left thalamus were positively correlated with adolescents’ scores on the Internet addiction scale. Altered regions involved in the limbic system (i.e., ACG, MCG, IPL, and thalamus) can lead to a decreased ability in information processing, particularly in monitoring and controlling personal behaviors and emotion. For example, the cingulate gyrus (CG) was involved in different stages of emotion processing, learning, memory, and executive functioning. Hence, the disruption of CG functioning may be related to problems in monitoring and controlling behaviors, specifically when these behaviors are driven by emotional states and reactions. Furthermore, the abnormal thalamo-cortical circuitry may be linked to attentional problems, which, together with impairment in response inhibition, may ultimately trigger problems in impulse regulation. Decreased connectivity in the group of Internet-addicted adolescents was also found in one intra-hemispheric connection, i.e., between the right caudate and the supra-marginal gyrus. At the same time, two inter-hemispheric connections (one between the left parietal lobe and the right frontal lobe, and another between the left occipital lobe and the right parietal lobe) exhibited increased connectivity in the Internet-addicted group. In general, deficits in long-range connections reflect a lack of integration process within networks and information processing, which are typical of the adult brain. In this sense, alterations are a form of deviation from the normal neurodevelopmental trajectory, and they are a common mark of different psychopathological conditions. In particular, the Internet-addicted group was characterized by larger clustering coefficients, indicative of the presence of relatively sparse long-distant and relatively dense short-distant functional connections, compared to controls.

Disrupted connectivity was also found in the study by Wang et al. (2017) involving 26 15-year-old adolescents with symptoms of Internet addiction and forty-three control participants. The authors focused on three main resting-state networks, i.e., the DMN, the CEN, and the SN. The group of Internet-addicted adolescents showed reduced functional connectivity in the DMN and a diminished interaction between the DMN and the SN. Abnormalities in the DMN may be related to a deficit in attentional orientation and self-referential processing, whereas reduced connectivity between the SN and DMN may be associated to a failure in suppressing internally oriented processing, thus decreasing the ability to control preoccupations and urges related to the Internet. Moreover, the Internet-addicted group reported a reduced inter-hemispheric functional connectivity of the right CEN, but also an increased intra-hemispheric functional connectivity of the left CEN. In general, the results of this study highlight the presence of a deficit in cognitive control, which are similar to the ones of other substance addictions. In particular, a decreased coherence of brain activity between CEN regions in different hemispheres was related to a deficit in executive functioning and control inhibition.

Another study by Chun et al. (2018) focused on both excessive smartphone use and Internet addiction as two forms of behavioral addictions. In a sample of 38 15-year-old participants and an equal number of control participants, they collected cortisol levels together with fMRI imaging data to investigate if addictive participants showed higher stress levels. Results highlighted that excessive smartphone users had diminished functional connectivity between the right OFC and NAcc and between the left OFC and the medial cingulate gyrus (MCC), which are regions related to cognitive control in the PFC and ventral striatum. In particular, the NAcc has been related to reward anticipation. Hence, it may modulate the learning process associated with the reward stimulus. The OFC is related to decision-making abilities (especially in reward-seeking behaviors), and in monitoring and evaluating reward outcomes. The authors found damaged connectivity between the PFC and the ventral striatum, which was found to be associated with impaired top–down executive control ability. Also, the authors found greater functional connectivity between the MCC and NAcc in adolescents that use the smartphone excessively. Since the MCC has the role of monitoring reward-related signals, it may be related to higher monitoring experiences based on reward expectancies. Withdrawal symptoms (including feelings of irritability and restlessness due to the interruption of the activity) were related to greater cortisol concentrations in excessive smartphone users for Internet-related activities compared to control participants. Hence, it is likely that cortisol secretion might affect frontostriatal connections afterward. The negative correlation between the frontostriatal connectivity and withdrawal symptoms can be explained by the fact that more developed cognitive inhibition abilities are related to less experience of these kind of symptoms.

#### Task-Related Paradigm

Two studies investigated the performance of adolescents with and without Internet addiction during a task-based fMRI acquisition. In order to investigate the difference in neural activity between 17 Internet-addicted adolescents and seventeen controls aged about 14 years, Kim et al. (2012) focused on disembodiment, i.e., the experience of feeling outside of one’s body, which may lead to dissociative processes and symptoms. The fMRI experimental session consisted of two fMRI tasks, self-agency on the throwing of a ball and the positioning of a ball. Both were presented to each participant with a block design. They were structured using the same animated configuration: three players (red, blue, and gray) arranged on the black background in a triangular distance from each other were throwing the ball. The first was “Task of the agency,” in which each participant had to click after the last player had caught the ball and throw it again to the other player. The second was the “Location task” in which each participant had to click when the ball was spatially between the two players. Different types of blocks were applied to give diverse points of view of the space between the players to each participant. In the Internet-addicted group, the authors found activation in the left middle temporal gyrus, the left middle occipital gyrus, the left thalamus, the left precentral gyrus, the right insula, and the right parahippocampal gyrus. In addition, fMRI results indicated that the duration of Internet use significantly correlated with the activity of the posterior area of the left middle temporal gyrus. Since their findings suggest a disembodiment-related activation of the brain in Internet-addicted adolescents, online behavioral addictions could be adversely related to brain development and the process of identity formation.

Li et al. (2014) investigated the neurobiological mechanisms of the lack of impulse control in relation to Internet addiction in a sample of 31 adolescents aged 15 years. During the fMRI session, they administered the Go-Stop paradigm, which is a task that allows evaluating the capacity to inhibit an initiated response. Eighteen Internet-addicted and 23 control participants were scanned, and the comparison of their fMRI results indicated that the indirect frontal-basal ganglia pathway was involved by response inhibition in the control group, but not in the Internet-addicted group. The latter group showed instead activation of the left superior frontal gyrus. Finally, they found a positive correlation between the percentage of successfully inhibited responses during the Go-Stop task and the strength of the connections from the IFG to the striatum as well as a negative correlation between the same behavior and the connection from the secondary visual cortex (V2) to the pre-supplementary motor area (pre-SMA), from the IFG to the pre-SMA, and from the pre-SMA to the IFG. Their results indicated a significant relationship between altered functional connectivity of brain areas involved during the response inhibition in adolescents with high levels of Internet addiction. The authors suggested that Internet-addicted adolescents showed a deficient response in inhibition because they most likely failed to recruit the neural correlates, which are usually involved during these cognitive processes.

## Discussion

**FIGURE 1:**
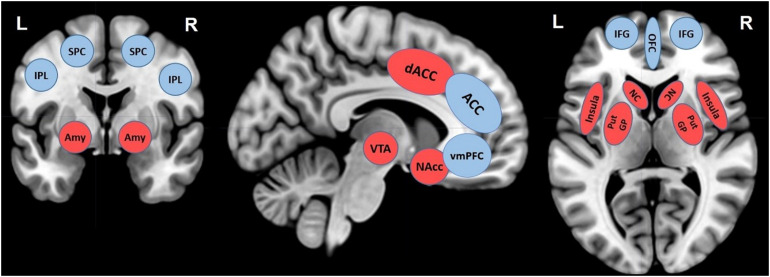
Overview of main brain regions reported in neuroimaging studies investigating structural and functional brain alterations associated with screen time in adolescence. The MRIcron software package (Rorden et al., 2007) was used to create brain renderings on a standard T1 template for illustrative purposes of brain areas involved in control (blue circles) and reward systems (red circles): ventro-medial prefrontal cortex (VMPFC), dorsal anterior cingulate cortex (dACC), anterior cingulate cortex (ACC), amygdala (Amy), putamen (Put), caudate nucleus (CN), inferior frontal gyrus (IFG), insula, nucleus accumbens (NAcc), ventral tegmental area (VTA), orbitofrontal cortex (OFC), superior parietal cortex (SPC), inferior parietal lobule (IPL). Lateralization, R, right; L, left.

Today’s adolescents live in a media-saturated world, with constant and easy access to gratifying and personalized contents on screen-based devices. Previous studies suggested small but negative relations between screen time and adolescent well-being (Orben, 2020), however, more and well-designed research that looks at the neural mechanisms involved in the effects of screen time, is still scarce. The present scoping review summarized the existing neuroscientific literature on the topic, with the aim to provide the first systematic and updated exploratory synthesis of the associations between screen time and adolescent brain development. This scoping review, including 16 original research papers, using task-unrelated and task-related neuroimaging paradigms published in the last 10 years, highlights important results and considerations which are summarized in Table 1 and Figure 1 and discussed hereafter.

**TABLE 1:** Summary of cortical and subcortical areas mainly involved in control and reward systems in the research on screen time in adolescence.

Brain areas	Functions
**Control system**	
Inferior frontal gyrus (IFG)	Motor control, working memory, language processing, empathy, imitative behaviors (mirror system) (Liakakis et al., 2011).
Inferior parietal lobule (IPL)	Self-perception (introspection and memory), social cognition, bottom–up attention (Igelström and Graziano, 2017).
Orbitofrontal cortex (OFC)	Decision making (monitoring, evaluation, anticipation of emotional and reward related behaviors – the somatic marker hypothesis), inhibition (e.g., delaying rewards), emotional processing (representations of rewards and punishments) (Kringelbach and Rolls, 2004; Kringelbach, 2005).
Ventromedial prefrontal cortex (VMPFC)	Emotional response and value, emotion regulation, fear conditioning, moral cognition, episodic and semantic memory, “affective meaning” (Young and Koenigs, 2007; Roy et al., 2012).
Anterior cingulate cortex (ACC) Superior parietal cortex (SPC)	Impact on cognitive control (Jaeger, 2013).
**Reward system**	
Dorsal anterior cingulate cortex (dACC)	Reward-based decision making (Bush et al., 2002)
Insula	Interoceptive/autonomic processing and integration of interoceptive sensory information, feeling of emotions, language (Critchley, 2004; Gasquoine, 2014).
Globus pallidus (GP) Caudate nucleus (CN) Putamen (Put) Nucleus accumbens (NAcc) Ventral tegmental area (VTA)	Reward processing (Galván, 2010; Silverman et al., 2015).
Amygdala (Amy)	Reward processing in variety of contexts (Wassum and Izquierdo, 2015)

The first key message of this review is that frequent and longer duration of screen-based media use (including Internet-related addictive behaviors) is related to a less efficient cognitive control system in adolescence. Indeed, stemming from studies on both general and clinical population, the frequency and amount of screen time, as well as Internet addiction, are related to reduced development of top-down cognitive control structures, both in terms of gray and white matter micro-structures. In particular, in line with other studies on (behavioral) addictions (Zhang and Volkow, 2019; Lüscher et al., 2020), including Internet Gaming Disorder (Meng et al., 2015; Yao et al., 2017), adolescents who spend more time on screens show reduced connectivity among subcortical, frontal, and parietal areas involved in attentional and control networks, both in terms of short- and medium-range connections. Abnormalities in these circuitries are generally related to attention problems and impairment in impulse regulation. Thus, considering the dual-systems model of the adolescent brain (Steinberg, 2010), adolescents who spend more time with screen media are more likely to experience difficulties in regulating their behaviors (e.g., sustaining attention, inhibitory control, planning), which increases impulsive tendencies (Sturman and Moghaddam, 2011; Casey and Caudle, 2013). Similarly, also adolescents who spend more time playing action video games have been reported as having problems in sustaining attention over time. These results can be interpreted considering that the online – and the game environment – provide new, stimulating, exciting, and attracting stimuli to the adolescent brain, thus making it more difficult to focus on more repetitive activities, requiring attention and planning, afterward (Trisolini et al., 2018). This is not only true for cold but also hot decisional processes. Indeed, the OFC/VMPFC are control areas relevant for emotional processes, and they have been constantly reported as altered in relation to frequent and compulsive digital media consumption. Interestingly, the interaction between hot and cold control systems was reported as pivotal in studying impulsivity and risk-taking behaviors in adolescence (McIlvain et al., 2020), hence future studies should be carried out focusing on the specific role of OFC/VMPFC in the context of digital media consumption. Connectivity within the DMN and the CEN, involving cortical and subcortical regions, were found to get stronger during early adolescent years (between 10 and 13), resulting in augmented segregation between the two functional networks (reflected by the greater anti-correlation over time). Additionally, the functional connectivity of the CEN correlates with IQ. Thus, individual differences in the structural and functional integration of frontoparietal regions are significant contributors to cognitive development, and early adolescence is a pivotal period during which screen-based media use can be related to activity in the CEN and the DMN.

The IFG, a region implicated in cognitive control and selection of competitive information and inhibition (Liakakis et al., 2011), was another area repeatedly found to be negatively associated to the consumption of screen-based devices. At the same time, the IPL (part of the DMN), which is related to different aspects of bottom–up attention (Igelström and Graziano, 2017), the OFC, which is involved in the different phases of the decision-making process including the (internal) representation of rewards (Kringelbach and Rolls, 2004; Kringelbach, 2005), and the ACC, which is a central region for cognitive control in general (Jaeger, 2013), have been constantly reported as less connected or developed in heavy adolescent media users. These areas are all involved in the implementation of goal-directed behaviors, which demand to control impulses and delay gratifications to optimize outcomes. This ability is supposed to mature through childhood and adolescence (Casey et al., 2008). Since, during adolescence, cognitive processes are still immature, frequent and longer screen time may potentially facilitate impulsive-like decisions and behaviors, especially considering that the limbic subcortical systems develop earlier with respect to the control system.

The second key message resulting from this review is that *online activities produce strong rewards for the brain, thus fostering subsequent online (addicted) behaviors to repeatedly seek short-term gratifications*. Adolescents already experience a motivational state valuing immediately available rewards more than long-term rewards (Sturman and Moghaddam, 2011; Albert et al., 2013), and the frequent and longer use of screen-based media can augment even more the tendency to seek short-term rewards. In particular, compared to children or adults, adolescents show greater activation of regions involved in reward anticipation (e.g., insula), indicating that they are particularly sensitive to the salience of the stimulus and they anticipate reward-based outcomes, also in terms of internal/visceral experiences (Silverman et al., 2015). At the same time, greater activation of regions related to the experience of the rewarding outcomes (e.g., putamen and amygdala), allow generating an emotive association with the outcome, which impacts future behaviors (Silverman et al., 2015). In line with these findings, Likes, which symbolize values of social appreciation and approval, act on the reward system in a similar way that making monetary contributions to others and providing social support do (Sherman et al., 2018a,b), which increments social bonds and directs further attentional processes. In adolescence, a period during which peer evaluation is pivotal, the presence of particularly rewarding cues, such as Likes and Followers on social media platforms, is experienced as very gratifying since social media platforms tackle the social sphere and create a new environment without limits in time and space, through which identity and social connections are further developed. The gratification obtained from social media platforms can also induce young people to spend more time in sedentary behaviors over physical activity, thus acting as a vicious circle and diminishing overall well-being as well as impulse control (Efraim et al., 2020). In addition, popular media contents (i.e., the ones with more Likes) were found to be rewarding despite the message they convey (e.g., also in case of risky and deviant contents). For this reason, popular screen-media contents may increase problematic behaviors, especially during adolescence, when top–down abilities are still underdeveloped, and the risk for psychological problems is higher (Sturman and Moghaddam, 2011). This may also easily lead to the experience of online risks, such as cyberbullying, which negatively impacts well-being over time (Camerini et al., 2020b; Marciano et al., 2020).

The two key messages are in line with the I-PACE model for addictive behaviors (Brand et al., 2019) and previous reviews on Internet addiction and Internet Gaming Disorder (Kuss and Griffiths, 2012; Brand et al., 2014; Meng et al., 2015; Yao et al., 2017), indicating that prefrontal dysfunctions are related to conflictual functioning between reward and control systems. Hence, in line with the I-PACE model, specific behaviors (e.g., Internet use, social networking) may relieve from negative moods. The associated subjective reward expectancies may change subsequent behavioral conducts, especially during adolescent years, since the brain is more sensitive to reward expectancies. This augments the probability to behave in the same way in similar situations, thus incrementing the urge of using screen-based media contents as a tool to avoid or face problems. As a consequence, screen time may influence how individuals cope with stressful situations, and this could be particularly true during the adolescent developmental period, during which coping strategies, such as problem-solving, increase but others, like escapism, decrease (Zimmer-Gembeck and Skinner, 2011). Besides maladaptive coping strategies, such as emotional suppression, denial, and avoidance, were associated with a higher risk for psychopathology in adolescence (Compas et al., 2017). Hence, the promotion of maladaptive coping through more frequent and more prolonged exposure to screen media can moderate the positive relationship between the consumption of media contents (as a way to deal with real-life stressors) and negative health outcomes that were widely reported in relation to media use in youth. This loop may also explain why control mechanisms become weaker and the cognitive and affective response gets increasingly guided by impulsive reactions.

The third and last key message of the present scoping review is that neuroscientific research on the impact of screen time on the adolescent brain is still at the beginning and there is a need for longitudinal studies and larger samples. Most of the summarized studies in this review used a cross-sectional design. Although “studying adolescence is like shooting at a moving target” (Sturman and Moghaddam, 2011, p. 1704), longitudinal studies or studies with an accelerated longitudinal design (Galbraith et al., 2017) are essential to track changes over time and disentangle causes and effects in this crucial developmental period. Furthermore, though cost- and time-intensive, fMRI studies should include larger sample sizes across the different age groups (ranging from pre-adolescence to late adolescence), thus allowing to look at more specific age-differences (Mills et al., 2014a) and consider subjective connections in different neural network developments (Mills et al., 2014b, 2016). In particular, studies focusing on Internet addiction focused on very small sample sizes, thus making it difficult to draw reliable conclusions. Additionally, all task-unrelated studies investigating Internet-related addictions came from Asian countries but two (Horowitz-Kraus and Hutton, 2018; Rodriguez-Ayllon et al., 2020), which were focused on general screen time. Thus, studies from other countries are warranted to draw reliable conclusions, which can be extended to different populations and cultures.

In addition, future neuroimaging studies should rely on detailed inclusion and exclusion criteria to aid the interpretation of their study findings. A more comprehensive evaluation of problematic Internet, social media, and smartphone use would provide further insights into their interrelation with brain functioning. To date, different scales are available [for a review of Internet addiction scales see Laconi et al. (2014) for the Bergen Social Media Addiction Scale see Duradoni et al. (2020); and for the smartphone addiction scales see Harris et al. (2020)], and their repeated use in empirical studies produces more robust findings on their psychometric properties and suitability. In a similar fashion, different and more nuanced measures of non-pathological screen time would overcome limitations of current assessments of screen time, which are often limited to overall frequency and duration of screen-media use as well as self-report data. Technological advancements now allow the integration of momentary ecological assessments of overall and content-specific screen-media use (Heron et al., 2017) as well as the collection of objective trace data (Stier et al., 2020) to overcome the current limits of self-reports (e.g., estimation bias, recall bias, social desirability bias; see also Slater, 2004) in the studies of neural correlates of screen time in adolescence. This should go along with a more comprehensive assessment of symptoms and psychological problems related to media use [including media-related constructs such as FoMO, social (appearance) anxiety, and cyberbullying], to decide on the eligibility for study participation or to control for these symptoms and problems in subsequent analyses. Additionally, future studies should improve their quality, also considering how experimental and control group are matched. For example, some studies failed in matching the experimental and control group with respect to age, gender, education, and IQ. In addition, the diagnosis of Internet addiction was sometimes based only on self-reports and not on a clinical interview. Furthermore, future research on screen time and brain functioning should investigate positive aspects of screen-media use, including Internet and social media use for building and maintaining social connections, social support, identity formation, as well as learning.

When it comes to the assessment of brain activities, there is still little knowledge regarding the role of some brain areas involved in emotion regulation and cognitive control processes that are likely to be related to (excessive) screen time. One such area is the VMPFC, a key area for communication during adolescence between the cortical and subcortical structures (Morese and Longobardi, 2020). At the same time, efficient connections with subcortical regions, including the hippocampus, amygdala, striatum, and VTA (Calabro et al., 2020), were found to be indispensable for cognitive control abilities, hence, a further look at how these connections develop and are shaped by different online activities is now needed. Finally, although we relied on the dual-process model, it has been seen as too simplistic (Pfeifer and Allen, 2012), hence further conceptual and more elaborated definitions of adolescents’ control processes should be considered in future research.

## Conclusion

The present scoping review is the first to systematically summarize neuroscientific evidence on the effects of screen-based media use on the adolescent brain. Even though this review did not consider gray literature and non-English publications, it identified crucial mechanisms through which screen time in adolescence is related to cognitive control processes and through which online activities turn into addictive online activities. This review also identified methodological gaps to be filled in future research applying a neuroscientific approach to allow a more in-depth understanding of the developing brain in the digital era.

## Author Contributions

LM contributed to developing the main research question, carrying out the literature search, collecting the included studies’ information, describing the results, and elaborating the discussion. A-LC contributed to developing the main research question, describing the results, and elaborating the discussion. RM contributed to developing the main research question, collecting the included studies’ information, describing the results, and elaborating the discussion. All the authors contributed to the article and approved the submitted version.

## Conflict of Interest

The authors declare that the research was conducted in the absence of any commercial or financial relationships that could be construed as a potential conflict of interest.

## Publisher’s Note

All claims expressed in this article are solely those of the authors and do not necessarily represent those of their affiliated organizations, or those of the publisher, the editors and the reviewers. Any product that may be evaluated in this article, or claim that may be made by its manufacturer, is not guaranteed or endorsed by the publisher.
