# Supplementary material for: The Developing Brain in the Digital Era: A Scoping Review of Structural and Functional Correlates of Screen Time in Adolescence
Source: Front Psychol. 2021 Aug 27;12:671817. doi: 10.3389/fpsyg.2021.671817 (PMC8432290; doi:10.3389/fpsyg.2021.671817)
Supplement: Supplementary file 1 [file Data_Sheet_1.docx]

**Appendix A**

List of keywords used for the systematic search: 

(TI) screen time OR screen-time OR digital media OR tablet* OR smartphone* OR cellphone OR iPad* OR computer OR internet OR social media OR SNS* OR Facebook OR Instagram OR SnapChat OR YouTube

 AND

(AB) brain OR fMRI OR neural OR cerebral OR functional magnetic resonance imaging OR structural magnetic resonance imaging OR BOLD OR structural imaging OR functional imaging

NOT

(AB) gam* OR gambl* OR brain-computer OR intervention

| Study ID | Country | Design | Sampling | Sample (EXP) | Male (%) | Age (M;SD or Range) | Sample (CTR) | Male (%) | Age (M;SD or Range | CTR group matched for | Exclusion criteria | Screen time construct | Measure of screen time | Brain outcome | Brief description of results |
| --- | --- | --- | --- | --- | --- | --- | --- | --- | --- | --- | --- | --- | --- | --- | --- |
| Chun 2018 | Korea | 2 | 0 | 38 | 84% | 14.90 (1.49) | 38 | 79% | 14.12 (1.34) | Gender, IQ | Presence of past or current major medical disorders (e.g., diabetes mellitus), neurological disorders (e.g., seizure disorders, head injury) or psychiatric disorders (e.g., majormood disorders). Not having a normal or corrected to- normal vision | Excessive smartphone use; problematic Internet use | Korean Smartphone Addiction Proneness Scale (SAPS) for Youth; Korean Internet Addiction Proneness Scale (the K-scale) | Resting-state functional connectivity (focused on the left OFC, right OFC, and midcingulate cortex (MCC), and the left and rightNAcc) | In the right hemisphere, the SP-addicted group showed weaker functional connectivity between the right OFC and the right NAcc, and between the left OFC and MCC. Whereas the connectivity was stronger between the right NAcc and MCC. Controlling for age, IQ, and depressive symptoms, functional connectivity between rOFC and rNAcc was weaker in the SP-addicted group, whereas it was stronger between between rNAcc and MCC. Withdrawal symptoms and cortisol concentrations was significantly correlated in adolescents with SP addiction. Internet use withdrawal symptoms was negatively correlated with the left frontostriatal connectivity in the adolescents with SP addiction. Left frontostriatal connectivity was negatively correlated with cortisol concentrations in adolescents with SP addiction. |
| Hong 2013b | Korea | 2 | 0 | 12 | 100% | 13.41 (2.31) | 11 | 100% | 14.81 (.087) | Gender, right-handed, IQ | Presence of neurological and psychiatric disorders | Internet addiction | YIAS | Resting-state functional connectivity | The IA group showed decreased connectivity in a network comprising 59 links (both short and long range connections), and involving 38 different brain regions. Reduced connection included subcortical regions - i.e. hippocampus, globus pallidus, and putamen- and frontal (around 24%) and parietal (around 27%) cortices. Bilateral putamen showed decreased connections with all three major cerebral lobes involved. No significant correlation between functional connectivity and YIAS score was found. |
| Hong 2013a | Korea | 2 | 0 | 15 | 100% | 13.33 (2.84) | 15 | 100% | 15.40 (1.24) | IQ | Presence of DSM -IV Axis I disorders including substance abuse, epilepsy or other neurological disorders, and past history of severe head trauma. | Internet addiction | YIAS | Cortical tickness | The thickness of lateral OFC was reduced in the IA group. The control group showed more cortical tickness in the OFC, the isthmus of the cingulate cortex, and the pars orbitalis in the right hemisphere and lateral occipital cortex in the left hemisphere |
| Horowitz-Kraus 2018 | USA | 0 | 0 | 19 | 42% | 9.99 (.084) | -- | -- | -- | -- | Low non-verbal intelligence quotient, history of neurological, emotional, or attention disorder; not being Eglish monolingual speakers, Caucasian, or with an average socioeconomic status | Duration of use (Smartphone, tablets, desktop/laptop, and television) | Ad hoc questions | Resting-state functional connectivity | The time spent reading positively correlated with higher functional connectivity between the visual word form area and regions related to language (Brodmann areas 40,42,43 and 22), visual association (Brodmann area 19), and cognitive-control (left Brodmann areas 7 and 44). Screen-time were negatively correlated with functional connectivity between the visual word form area, the regions related to cognitive control (right Brodmann areas 24 ,13, and left 25 and 47), and language regions (right Brodmann areas 39 and 20). All the results were controlled for reading skills. |
| Lin 2012 | China | 2 | 2 | 17 | 88% | 17.01 (2.50) | 16 | 88% | 17.78 (2.46) | Age, gender years of education | History of: substance abuse or dependence, major psychiatric disorders, such as schizophrenia, depression, anxiety disorder, psychotic episodes, or hospitalization for psychiatric disorders. Presence of ongoing medications. | Internet addiction | YDQ | Whole brain voxel-wise analysis of fractional anisotropy (FA) | Compared to the control subjects, IA subjects had significantly reduced FA in bilateral orbito-frontal white matter, corpus callosum, association fibers with the involvement of bilateral inferior front-occipital fasciculus and the bilateral anterior cingulum, projection fibers consisting of the bilateral anterior, superior, and posterior corona radiation, bilateral anterior limb of the internal capsule, bilateral external capsule, and left precentral gyrus. There were no white matter regions where the controls had significantly lower FA values compared with the IA participants. |
| Rodriguez-Ayllon 2020 | Netherlands | 0 | 1 (Generation R study participants) | 2532 | 50% | 10.12 (.58) | -- | -- | -- | -- | - | Frequency and duration during weekdays and weekend days (Television, computer, video-games) | Ad hoc questions | White matter miscrostructure - FA and MD (DTI) | Controlling for sex, age, ancestral background, BMI, maternal education, emotional and behavioural problems and non-verbal IQ, physical activity (sport participation in particular) was positive associated with FA. Physical activity (outdoor playing and sport aprticipation in particular) was negatively associated with MD. Screen-time was not significantly associated with any global DTI metrics. |
| Takeuchi 2018 | Japan | 0;1 (36 months) | 0 | CS: 284; L=223 | CS: 49%; L:52% | CS: 11.2 (3.1); L: 14.2 (3.0) | -- | -- | -- | -- | Intelligent quotient less then 80 | Frequency of Internet use | Ad hoc questions | Grey and white matter volume (VBM) | CS: the frequency of Internet use did not significantly correlate with any rGMV or rWMV measure or with the intelligence tests. L: After correcting for confounding variables, the frequency of internet use at T1 showed a significant negative correlation with the change in rGMV of a widespread anatomical cluster at T2, including extensive bilateral perisylvian areas, the bilateral temporal pole, the bilateral cerebellum, bilateral medial temporal lobe structures (hippocampus and amygdala), bilateral basal ganglia structures, the bilateral inferior temporal lobe, the thalamus, the bilateral orbitofrontal gyrus and lateral prefrontal cortex, the insula, and the left lingual gyrus. Changes included also the WM adjacent areas. The frequency of internet use at T1 negatively correlated with the change in the Verbal Intelligence Quotient. |
| Wang 2017 | China | 2 | 2 | 26 | 68% | 15 (1.3) | 43 | 70% | 15.1 (.5) | Age, gender | Presence of DSM-IV axis I disorder, including substance abuse disorder | Internet addiction | YDQ | Resting-state functional connectivity (focused on DMN, FPN, SN) | The IA group showed reduced inter-hemispheric functional connectivity of the right FPN (IPL), increased intra-hemispheric functional connectivity of the left FPN (between the inferior frontal gyrus and angular gyrus), reduced functional connectivity in the DMN (including the dorsal medial prefrontal cortex), and decreased interactions between the SN and anterior DMN. |
| Wee 2014 | China | 2 | 2 | 17 | 88% | 17.3 (2.6) | 16 | 88% | 17.7 (2.5) | Right-handed, age, gender, education | History of: comorbid psychiatric and non-psychiatric disorders, such as anxiety disorder, depression, compulsivity, schizophrenia, autism, or bipolar disorder; substance abuse or dependency; physical disorders related to the motion, digestive, nervous, respiratory, circulation, endocrine, urinary, and reproductive systems; and pregnancy or menstrual period in women during the day of scanning. | Internet addiction | YDQ | Resting-state functional connectivity (using a graph theoretical approach) | IAD patients showed nodal centrality alterations predominantly located in the left inferior parietal lobule (IPL), right anterior cingulate gyrus (ACG) - part of the DMN - left thalamus (THA), and regions of the the limbic system (e.g., right middle cingulate gyrus (MCG)). ACG, MCG, and THA were also positively correlated with IA scores. Two inter-hemispheric connections, one between the parietal and frontal lobe and one between the occipital and the parietal lobe, exhibit increased connectivity in the IA group. One intra-hemispheric connection, between the right caudate and the right parietal lobe, shows decreased connectivity in the IA group. |
| Zhou 2011 | China | 2 | 2 | 18 | 89% | 17.23 (2.60) | 15 | 87% | 17.81 (2.58) | Age, gender; no personal or family history for psychiatric disorders; right handed | -- | Internet addiction | YDQ | Gray matter density - GMD (VBM) | The IA group showed lowerGMD in the left anterior cingulate cortex (ACC), left posterior cingulate cortex (PCC), left insula, and left lingulate gyrus, all areas responsible for modulating emotional behavior. No significant difference was found in the white matter change between the two groups. |

Table 2. Summary of the studies based on resting state fMRI and VBM. Legend. Design: 0=cross-sectional, 1=longitudinal, 2=control trial; Sampling: 0=convenient, 1=random, 2=outpatients; CS= cross-sectional, L=longitudinal; IA= Internet Addiction, SP= smartphone

| ID | Country | Design | Sampling | Sample (EXP) | Male (%) | Age (M; SD or Range) | Sample size (CTR) | Male (%) | Age (M; SD or Range) | CTR group matched for | Exclusion criteria | Screen time construct | Tasks | Brief task description | Brief description of results |
| --- | --- | --- | --- | --- | --- | --- | --- | --- | --- | --- | --- | --- | --- | --- | --- |
| Efraim 2020 | USA | 2 | 1 | 32 | 63% | 8.7 (0.5) | -- | -- | -- | -- | Not having normal or corrected to normal vision, not qualified to participate in strenuous physical activity, presence of current orthopedic impairments based on the physical activity readiness questionnaire, History of neurological disorder, and previous incident of traumatic brain injury. | Frequency of smartphone use | Participants engaged in an active and passive condition, after which they perform a go/no-go task | Task involves the presentation of high- and low-calorie food pictures | There was no main effect for condition (active vs. sedentary) observed for any region of the brain in either the a priori regional or exploratory whole brain analyses. The a priori ROI analysis showed a significant stimulus (high- vs. low-calorie) by condition interaction in the right superior parietal cortex, right post-central gyrus, NAcc and bilaterally in the ACC. However, after correcting for multiple comparisons only the left ACC and the right superior parietal cortex met the adjusted critical value. There was also a significant main effect of picture type, bilaterally, in the lateral OFC. The whole brain analysis did not reveal any regions of activation nor any significant main effect for picture type after controlling for multiple comparisons. Significant stimulus by condition interactions were found in the right superior parietal cortex, and left ACC. High-calorie pictures elicited significantly more activation bilaterally in the OFC compared to low-calorie pictures. |
| Kim 2012 | Korea | 2 | 0 | 17 | 100% | 13.76 (.83) | 17 | 100% | 13.76 (.83) | Gender , right-handed, IQ | Score less than 80 on the short-form of theWechsler Intelligence Scale for Children; History of head injuries or other major neurologicaldisorders; Presence of medical or surgical illnesses; Current diagnoses as schizophrenia, major depression with psychoticfeatures, bipolar disorder, or substance use disorder | Internet addiction | Agency Task; Location Task | Three players (red, blue, and gray), in a triangular arrangementin a black background, throw a ball to each other. For the Agency Task was required to click the mouse after the gray player caught the ball and before to threw it to the other player. For the Location Task was required to click the mouse when the ball was mid-air between the two players. | Activations for the IA group were observed in the leftprecentral gyrus (BA 44), the right precentral gyrus (BA 6), the leftmiddle frontal gyrus (BA 6), the right middle frontal gyrus (BA 46), the left thalamus, the left cuneus (BA 18), the left postcentral gyrus(BA 5), and the right inferior parietal lobule (BA 40). In contrast, no activation was observed in the control group, butactivation was observed in the right paracentral lobule (BA 31) when the threshold was raised to pb 0.001. IA group compared to control group exhibited higher activation in the posterior area of the left middle temporal gyrus, the left middle occipital gyrus (BA 19), the left thalamus, the leftprecentral gyrus (BA 3), the right insula (BA 13), and the right para-hippocampal gyrus (BA 19). In the IA group, both the duration of Internet use and the degree of IA were significantly correlated with the brain activity of disembodiment-related areas, which are pivotal in the identity formation. |
| Li 2014 | China | 2 | 2 | 18 | n.a. | 15.1 (1.4) | 23 | n.a | 15.2 (0.5) | Age, ethnicity, education | Presence of DSM-IV axis I disorders | Internet addiction | Go-Stop paradigm | This paradigm was developed to assess the inhibition response, that has already been initiated, to a series of five-digit numbers. | The control group showed activation in the indirect frontal-basal ganglia pathway (including connectivity between IFG, pre-SMA and pre-SMA and striatum, IFG and striatum) during response inhibition, whereas the IA group did not (and showed increased activation in left superior frontal gyrus), resulting in failing to recruit this pathway and inhibit unwanted actions. IA group compared to the control group showed activations in bilateral IFG and the right striatum. |
| Sherman 2016 | USA | 0 | 1 | 34 | 47% | 13-18 | -- | -- | -- | -- | -- | Providing Likes on social media | Instagram task | The task involved the presentation of photos, accompanied by text indicating how many other people had already liked the image; the participant had to choose “Like” or not. | Viewing photos with many (compared with few) likes was associated with greater activity in neural regions implicated in reward processing, social cognition, imitation, and attention. In addition, when adolescents viewed risky photos (as opposed to neutral photos), activation in the cognitive-control network decreased. ROI analyses showed significantly greater activation in the bilateral NAcc, in the left NAcc when participants viewed neutral images that had many likes than when they viewed neutral images that had few likes. |
| Sherman, Greenfield, and colleagues (2018a) | USA | 0 | 1 | 61 | 41% | 18.2 (13-21) | -- | -- | -- | -- | History of developmental, psychiatric, or neurological disorders. | Providing Likes on social media | Instagram task | The task involved the presentation of photos, accompanied by text indicating how many other people had already liked the image; the participant had to choose “Like” or not. | High school and college students did not significantly differ in activation in left or right NAcc when they received many Likes compared to few. Similarly, the cohorts did not differ in NAcc response when viewing popular (compared to unpopular) risky or neutral images. Instead, college students, during the view of popular than unpopular risky images activated significantly the left NAcc. The experience of providing Likes to others on social media, as well as receiving Likes from others, was related to activation in brain circuity implicated in reward system (including the striatum and ventral tegmental area). |
| Sherman, Hernandez, and colleagues (2018b) | USA | 0 | 1 | 58 | 41% | 18.2 (13-21) | -- | -- | -- | -- | Presence of neurological, psychiatric, or developmental diagnosis or MRI contraindications | Providing Likes on social media | Instagram task | The task involved the presentation of photos, accompanied by text indicating how many other people had already liked the image; the participant had to choose “Like” or not. | Like>Next contrast was associated with greater activation in the dorsal striatum (caudate andputamen),thalamus, bilateral insula/orbitofrontalcortex,hippocampus, amygdala, a considerable portion of the anteriorcingulate and paracingulate cortex, inferior frontal gyrus and the bilateral intraparietal sulcus. When participants Liked an image, they showed activation in the ventral striatum and vmPFC, related to regions associated with reward processing and prosocial behavior. Experience of providing Likes to others on social media related to activation in brain circuity implicated in reward, including the striatum and ventral tegmental area, regions also implicated in the experience of receiving Likesfrom others. |

Table 3. Summary of the studies fMRI task-based. Legend. Design: 0=cross-sectional, 1=longitudinal, 2=control trial; Sampling: 0=convenient, 1=random, 2=outpatients; IA= Internet Addiction
